# Contribution of historical herbarium small RNAs to the reconstruction of a cassava mosaic geminivirus evolutionary history

**DOI:** 10.1038/s41598-021-00518-w

**Published:** 2021-10-28

**Authors:** Adrien Rieux, Paola Campos, Arnaud Duvermy, Sarah Scussel, Darren Martin, Myriam Gaudeul, Pierre Lefeuvre, Nathalie Becker, Jean-Michel Lett

**Affiliations:** 1 CIRAD, UMR PVBMT, 97410 St Pierre, La Réunion, France; 2 Institut de Systématique, Evolution, Biodiversité (ISYEB), Muséum national d’Histoire naturelle, CNRS, Sorbonne Université, EPHE, Université des Antilles, 57 Rue Cuvier, CP 50, 75005 Paris, France; 3 Computational Biology Division, Department of Integrative Biomedical Sciences, Institute of Infectious Diseases and Molecular Medicine, University of Cape Town, Observatory, Cape Town, South Africa; 4 Herbier national (P), Muséum national d’Histoire Naturelle, CP39, 57 Rue Cuvier, 75005 Paris, France

**Keywords:** Computational biology and bioinformatics, Molecular ecology

## Abstract

Emerging viral diseases of plants are recognised as a growing threat to global food security. However, little is known about the evolutionary processes and ecological factors underlying the emergence and success of viruses that have caused past epidemics. With technological advances in the field of ancient genomics, it is now possible to sequence historical genomes to provide a better understanding of viral plant disease emergence and pathogen evolutionary history. In this context, herbarium specimens represent a valuable source of dated and preserved material. We report here the first historical genome of a crop pathogen DNA virus, a 90-year-old African cassava mosaic virus (ACMV), reconstructed from small RNA sequences bearing hallmarks of small interfering RNAs. Relative to tip-calibrated dating inferences using only modern data, those performed with the historical genome yielded both molecular evolution rate estimates that were significantly lower, and lineage divergence times that were significantly older. Crucially, divergence times estimated without the historical genome appeared in discordance with both historical disease reports and the existence of the historical genome itself. In conclusion, our study reports an updated time-frame for the history and evolution of ACMV and illustrates how the study of crop viral diseases could benefit from natural history collections.

## Introduction

Crop pests and diseases have plagued farmers since the dawn of agriculture^1^. Today they continue to be major threats to agro-ecosystems worldwide, significantly reducing yields, incurring economic losses and threatening food security^2,3^. Amongst the different taxonomic groups of crop pathogens, viruses account for almost half of emerging infectious diseases^4^ and, as such, are a major focus of ongoing scientific investigation^5^.

The effective management of infectious viral crop diseases requires understanding the factors underlying virus emergence, adaptation and spread^6^. Elucidating the history of a pathogen’s emergence is a prerequisite to inferring the evolutionary, ecological and anthropogenic factors that have driven the past epidemiological dynamics of the pathogen; inferences which could in turn be used to design efficient future disease control strategies^7^. As sequencing technologies have become more accessible, pathogen genomic analyses have played an increasingly important role in infectious disease research^8^. Concomitantly, recent methodological developments in molecular phylogeography can now be applied to study the emergence and evolution of viral pathogens in space and time with an unprecedented degree of accuracy^9^. Examples of such recent inferences performed on field-sampled viruses include the reconstruction of the spread and evolution of tomato yellow leaf curl virus (TYLCV) ^10^, maize streak virus (MSV)^11^ or rice yellow mottle virus (RYMV)^12,13^. Interestingly, analyses of ancient DNA and RNA virus genomic sequence data obtained from herbaria or archaeological material have demonstrated that historical samples can be leveraged to substantially improve phylogenetic based molecular dating studies^14–16^. By countering the molecular-clock calibration biases that occur when using modern genomes to infer ancient lineage divergence times, the addition of ancient genomes with known sampling dates commonly yields estimates of viral lineage divergence times that are older and more in accordance with historical reports than when the ancient sequences are not included in molecular dating studies^17,18^. In this context, the oldest historical crop-associated virus genome published to date is a member of the *Chrysovirus* genus isolated from a 1,000 year old maize cob^19^.

High throughput sequencing (HTS) and bioinformatic analyses have already contributed to a paradigm shift in the fields of virus discovery and diagnosis^20–22^. Among various possible targets, such as virion-associated nucleic acids, double-stranded RNAs, total RNAs, ribosomal-RNA-depleted RNAs or messenger RNAs, the sequencing of small RNAs (sRNA) offers several advantages^23^. First, since plant viruses are targeted by host silencing mechanisms, the sequencing of small interfering RNAs (siRNAs) should enable the identification of all types of plant viral agents, whatever the nature or structure of their genomes (DNA or RNA, single or double stranded). In this context, the pioneering work of Kreuze et al.^24^ demonstrated the universal power of targeting, sequencing and analysing sRNAs for the comprehensive reconstruction of viral genomes from fresh material of cultivated and non-cultivated plants (as reviewed in^25^). Moreover, viral sRNAs were reported as more stable than long RNA and DNA molecules, and proved to be suitable for deep sequencing, including paleovirology applications for several plant RNA phytoviruses^17,26^. As an illustration, Smith et al.^17^ have reported the identification and reconstruction of an ancient isolate of barley stripe mosaic virus (Genus *Hordeivirus*, family *Virgaviridae*) by sequencing sRNAs extracted from 700 years-old barley seeds, with 99.4% of the contemporary virus reference genome being covered by sRNA contigs. In a recent study reconstructing RNA phytovirus genomes, a detailed characterisation (using size distribution and coverage data) underscored the preservation of siRNAs among viral sRNAs from dried, modern samples, yet to be shown from historical samples^27^.

Cassava cultivation is associated with a wide range of diseases that seriously undermine the food and economic security in sub-Saharan African countries, the most notable of which is cassava mosaic disease (CMD), caused by a complex of cassava mosaic geminiviruses (CMGs, genus *Begomovirus*, family *Geminiviridae*)^28^. CMD is currently the most damaging plant virus disease in the world (estimates of US$1.9–2.7 billion annual loss) and was associated with an East African famine in the late 1990s that likely caused the deaths of thousands of people^29^. As an expanding global threat, CMD is currently under surveillance in Southeast Asia since its first description in Eastern Cambodia in 2016^30,31^. CMGs are transmitted by whiteflies of the *Bemisia tabaci* species complex or by the use of infected cuttings (for review see^28^). In sub-Saharan Africa cassava growing areas, several native species of the *B. tabaci* species complex referred as sub-Saharan African species (SSA) have been reported as the prevalent whiteflies associated with the spread of viruses that cause cassava mosaic disease (CMD)^32^. However, several cassava surveys suggest that the use of infected cassava cuttings for the establishment of new plantations appears to be largely responsible for the high incidence of CMD in sub-Saharan Africa^33,34^. CMGs possess bipartite genomes, with genome components, called DNA-A and DNA-B, comprising 2.7 kb circular single-stranded DNA molecules. Both components are necessary for successful infection of cassava. While DNA-A encodes proteins and regulatory elements responsible for replication, encapsidation functions and the control of gene expression, DNA-B encodes proteins enabling viral movement^35^. In plant cells infected by geminiviruses, bidirectional read-through transcription of the circular viral dsDNA generates sense and antisense transcripts^26^. These dsRNA overlapping transcripts are processed by Dicer-like (DCL) family proteins from the RNA interference machinery, generating 21, 22 and 24 nt siRNAs and covering the entire circular virus genome (including coding sequences, as well as the intergenic region that contains the promoter^36,37^).

Interestingly, whereas cassava originates from South America^38^, the African CMGs are endemic to Africa and are likely recent descendants of geminiviruses adapted to infect indigenous uncultivated African plant species^39^. Therefore the adaptation of CMGs to cassava could have only started, either after cassava was introduced to West Africa in the Gulf of Guinea during the 16th century, or after it was introduced to East Africa and the South West Indian Ocean islands in the 18th century. Since the initial characterisation in the early 1980s of the first known CMG species, African cassava mosaic virus (ACMV), several others have been described in sub-Saharan Africa, surrounding islands and the Indian subcontinent^40^. The distribution of ACMV on the African continent has enabled the use of phylogeographic studies to investigate its evolutionary and epidemiological dynamics. Based on time-scaled phylogeographic analyses of modern ACMV isolates sampled between 1982 and 2012, it has been inferred that ACMV-driven CMD began disseminating in the 1980s only, with a single discreet movement of the virus from East Africa to Madagascar between 1996 and 2003^41^.

Here we report the genome of a 90-year-old ACMV isolate reconstructed from sRNAs, characteristic of *bona fide* siRNAs and whose damage patterns prove its authenticity. Using tip-calibrated phylogenetic inferences, we estimate both rates of molecular evolution and divergence times, underscoring the contribution of the historical genome in this calculation. Finally, we demonstrate how this single genome significantly improves our understanding of the history of ACMV in Africa.

## Results and discussion

### Nucleic acids isolation and high-throughput sequencing

**Figure 1:**
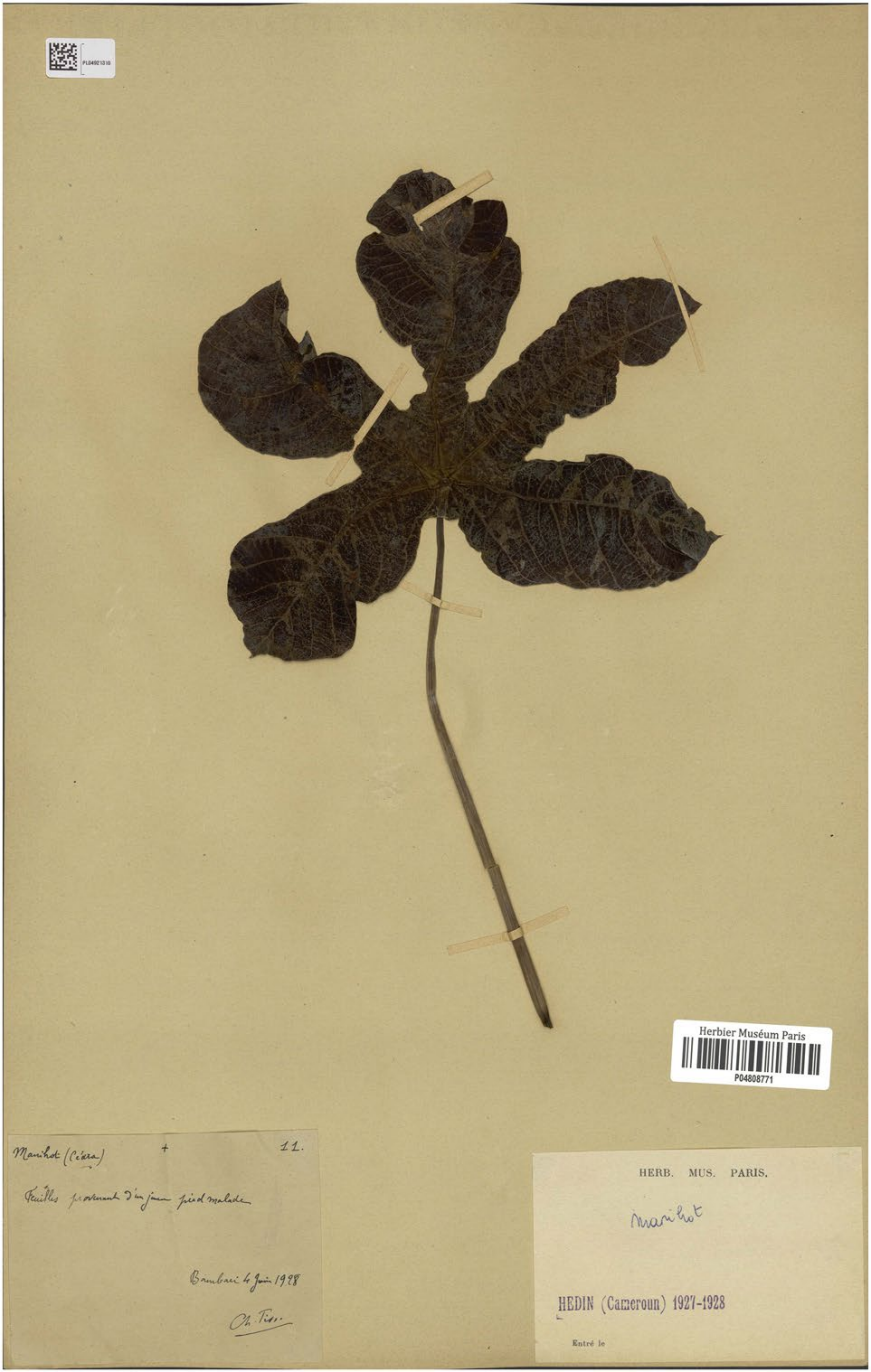
Leaf of *Manihot glaziovii* specimen P04808771 collected in Bambari, Central African Republic, in June 1928 and preserved at the Herbarium of the Muséum national d'Histoire naturelle, Paris, France. The original annotation (hand-written in French on bottom left) states ”Leaf from a young diseased plant”. Typical symptoms of cassava mosaic disease such as chlorotic mosaic and deformation of the leaf can be distinguished.

**Figure 2:**
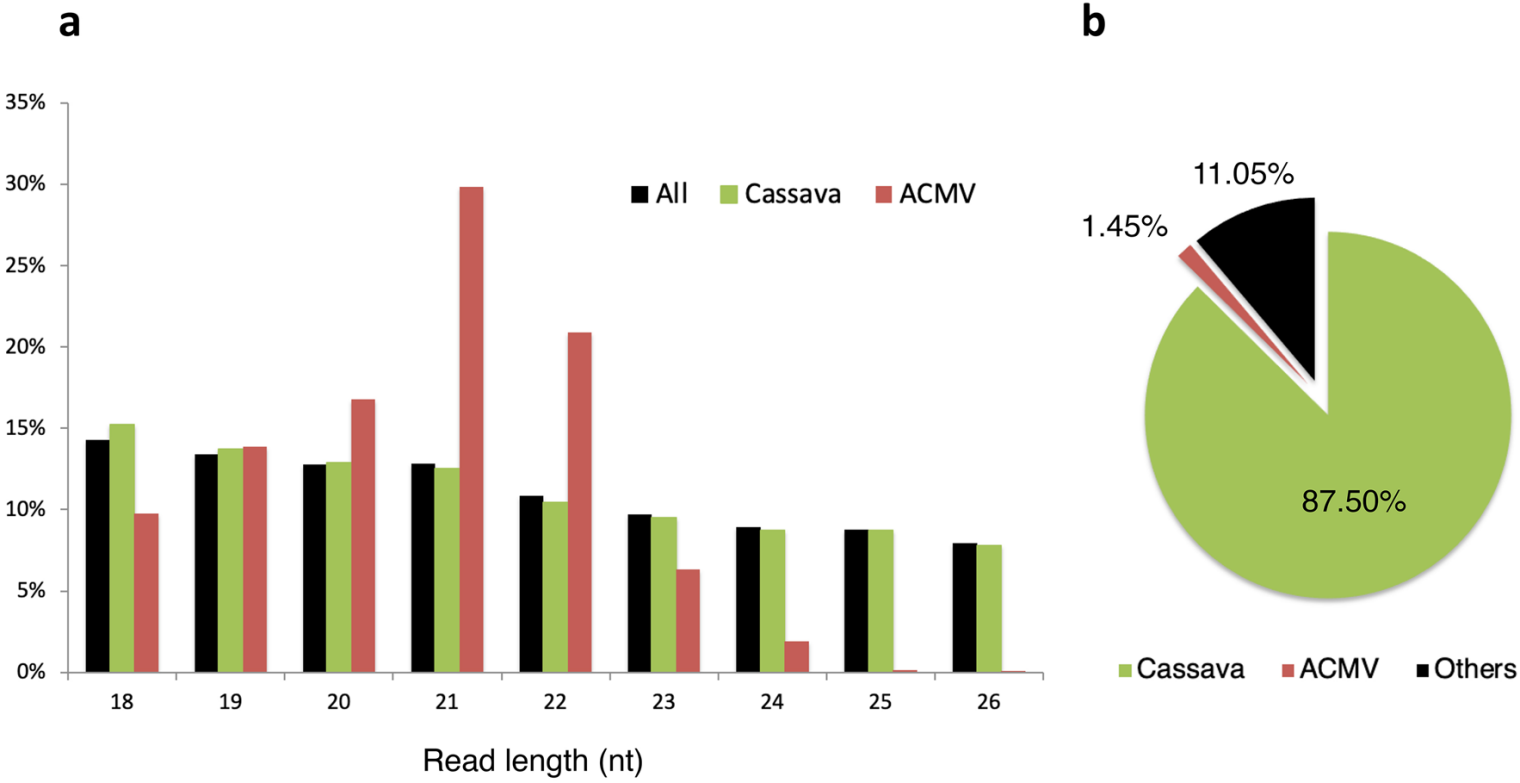
Main characteristics of small RNA (sRNA) isolated from historical specimen P04808771 (Herbarium of the Muséum national d'Histoire naturelle, Paris, France). (**a**) Size distribution of all, cassava-mapping and ACMV (DNA-A & DNA-B) genome-mapping reads. (**b**) Proportion of reads mapping to cassava and ACMV reference genomes.

From a small leaf fragment of a *Manihot glaziovii* (cassava) herbarium leaf specimen (Fig. 1) collected in the Central African Republic in 1928 and displaying typical symptoms of CMD, 185 ng of total DNA and 215 ng of total RNA were carefully extracted in a bleach-cleaned hospital laboratory with no prior exposure to plant material. Our first attempt to amplify and sequence viral DNA following Rolling Circle Amplification (RCA) failed (data not shown), likely due to substantial fragmentation of DNA, as previously described for herbarium specimens of similar age^42^ . Hence, based on the pioneering work of Kreuze et al.^24^ and Smith et al.^17^, we decided to target sRNAs. After library construction, high throughput sequencing of the sRNA fraction on an Illumina Hi-Seq High Output platform generated 8.6 M single-end reads with a base call accuracy of 99.90 to 99.96%. Following adaptor trimming and quality checking, reads ranging from 18 to 26 nt in length were selected for further analyses (Fig. 2a).

### Detection of genuine historical ACMV in herbarium cassava specimen

The analysis of sRNA reads with VirusDetect software revealed the presence of both DNA-A and DNA-B ACMV segments within the historical cassava specimen, with one contig covering 99.3% of the reference DNA-A sequence and fourteen contigs covering 88.7 % of the reference DNA-B sequence (Figure S1). We hence attempted to PCR amplify ACMV-specific DNA but no amplicons were successfully generated (data not shown). This result further highlights the promising potential underlying sRNAs sequencing to reconstruct historical viral pathogen genomes, as compared to classical approaches targeting DNA. Both DNA-A and DNA-B contigs harboured all eight typical open reading frames (ORFs) and inverted repeats (IRs) described for bipartite cassava geminiviruses (as depicted in^43^). No other viruses were detected by VirusDetect from this sample. Running BWA-aln, a dedicated tool optimised for small read mapping, 1.45% of reads aligned to ACMV reference sequences and 87.55% of reads mapped to the *M.glaziovii* (cassava) reference sequence (Fig. 2b). Interestingly, among the 18–26 nt sRNAs mapping to ACMV or cassava, a predominance of ACMV-mapping sRNAs was observed for 21 and 22 nt sRNAs (Fig. 2a). These viral sRNAs may represent siRNAs, among the 21, 22 and 24 nt siRNA size classes described for geminiviruses^25^.

**Figure 3:**
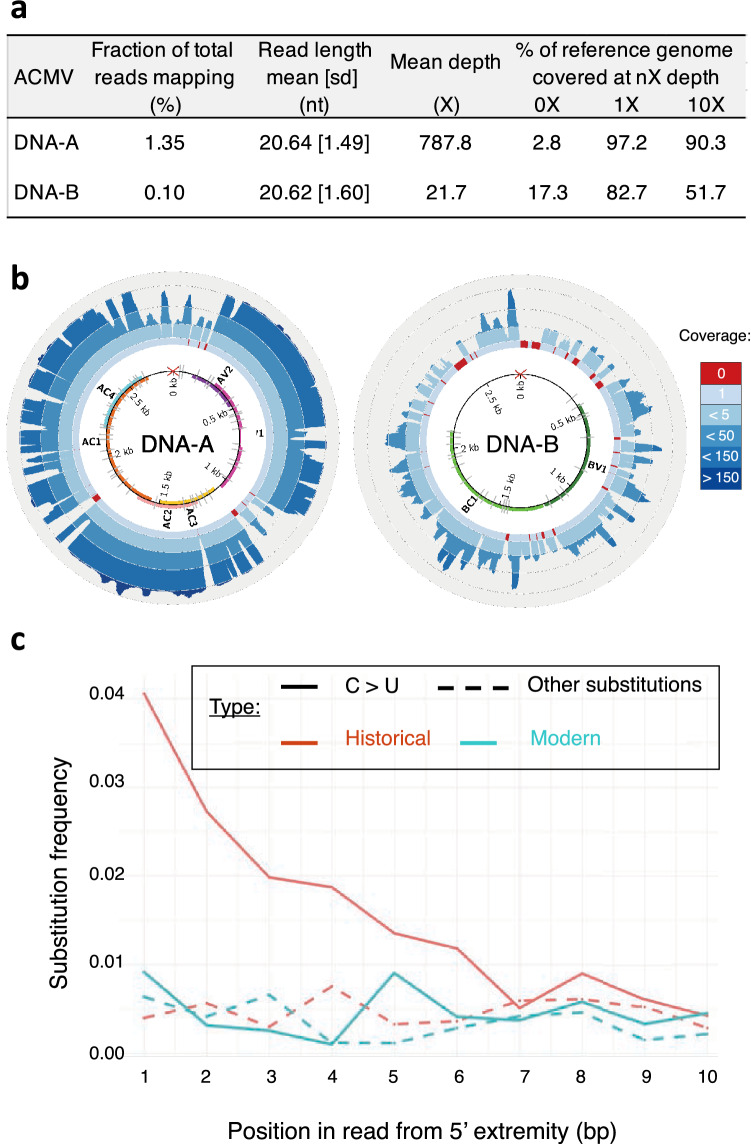
Reconstruction and authentication of historical ACMV genome. (**a**) Summary of mapping statistics to reference genomes for both ACMV DNA-A and DNA-B molecules. (**b**) Coverage plots (blue scale). Red arrays indicate regions that are not covered with siRNA reads (depth=0). Inner circle represents the genome and coding regions, as follows: AC1, AC3, AC3, AC4, AV1 and AV2 for DNA-A ; BC1 and BV1 for DNA-B; C: complementary strand; V: viral strand. Red cross symbolizes the geminivirus replication initiation site and grey ticks the SNPs between historical and reference sequences. (**c**) Post-mortem RNA damage patterns measured on historical (red) and modern ACMV sample isolated in 2017 (green). Straight and dotted lines represent C to U *vs* all other substitutions of the first 10 nucleotides from the 5’end, respectively.

To authenticate the historical nature of the ACMV siRNA reads and rule out the possibility of them being derived from lab contaminations, they were assessed for the presence of postmortem RNA damage. We found a clear pattern of C to U deamination reaching maximum values (±4%) at read extremities and declining exponentially inwards (Fig. 3C), as expected and previously shown for historical RNA^17,44^. In addition, the examined modern control displayed no such pattern. We found no difference in deamination patterns between DNA-A and DNA-B segments (not shown). The historical consensus sequences of ACMV DNA-A and DNA-B were reconstructed and covered 97.2% and 82.7% of the reference sequences (at 1X-fold) with a mean depth of 787.8 and 21.7 fold, respectively (Fig. 3A, B). Importantly, our mapping strategy aiming to reconstruct ACMV DNA-A and DNA-B consensus sequences was shown robust to (i) the choice of the short-read aligner, (ii) the presence of shared genomic regions between DNA-A & DNA-B segments and (iii) the choice of the reference sequences (Figure S2). The difference in sequencing depth between DNA-A and DNA-B could be explained by a difference in the abundance of these components in the plant tissues, and/or by higher host plant's RNAi-based antiviral defences targeting the DNA-A. In line with this latter observation, analyses of siRNA in ACMV-infected plants (*Nicotiana benthiamana* and cassava)^36,43^ showed a majority of siRNAs derived from the DNA-A component. A more detailed analysis of sRNA read coverage (Figure S3) locates a hotspot on ACMV-A, corresponding to overlapping transcripts coding for AC1, AC2 and AC3, consistent with previous siRNA analyses derived from ACMV^36,43^.

Recent large-scale surveys have revealed pervasiveness of transcriptionally active endogenous geminiviral sequences (EGSs) in several plant genomes^45,46^. The hypothetical presence of sRNAs deriving from EGVs and their use in our analyses could potentially impact the reconstruction of our ancient viral sequences. However, for all the arguments developed below, we are convinced that the sRNAs sequenced in this study are from episomal viral DNA rather than EGS. First, to date only small portions of endogenous geminiviral sequences were proposed to be transcribed (homologous to ren and rep genes^45,46^) while we were able to reconstruct a nearly complete ACMV genome from sRNA sequences. Second, in their recent study, Sharma et al.^46^ did not find any trace of EGSs within the genome of *Manihot esculenta*. In this work, we analysed the currently publicly available genomic resources of *Manihot glaziovii*^47^. Importantly, of the contigs that displayed similarities with geminiviruses (of length ranging between 163 and 2929 nt), all the hits covered 99 to 100% of the contigs. No chimeric contigs (containing both viruses and cassava sequences, that would indicate the presence of EGS), were detected (Table S3). This observation suggests that the analysed *M. glaziovii* genomes were generated from plants contaminated with episomal geminiviruses. Third, the herbarium specimen analysed displayed typical symptoms of Cassava Mosaic Disease. Although symptoms promoted by integrated viral sequences are theoretically possible, they wouldn't be expected for endogenous virus sequences, whose partial integration is unlikely to promote any infection^46^, even for the longest integrated EGSs described so far^45^. In addition, geminiviral endogenous elements have not so far been reported to give rise to episomal viruses^25,48^. Finally, our reconstructed ACMV genomes showed a very high pairwise genetic identity (>99%) with their modern counterparts, a value that we would predict to be smaller in case of non-functional geminivirus sequences integrated in plant genomes for long periods^49^.

### Phylogenetic inferences and dating using both historical and modern sequences

In order to investigate the phylogenetic relationship of our historical sequences to those already available from recent samples, we built nucleotide alignments of our historical genome along with 134 and 99 public modern African ACMV DNA-A and DNA-B sequences, respectively. The historical and modern sequences displayed an average nucleotide divergence of 2.3% for DNA-A and 2.9% for DNA-B. Two recombinant events were detected in the ACMV sequences analysed in this study (Table S1). Recombinant ACMV regions (positions 631-781 & 1901-1933 relative to AY211884 sequence for ACMV DNA-A) were identified with RDP4^50^ and removed from further inferences to avoid the potentially confounding effects these could have on the accuracy of inferred phylogenies. Note that as a precaution, recombinant region 2 was removed from the analysis, despite being detected in the historical DNA-A sequence with a single method only. The 1081 and 850 non-recombining SNPs obtained for ACMV DNA-A and DNA-B respectively were used to build Maximum-Likelihood (ML) phylogenies, using a cassava mosaic Madagascar virus (CMMGV) isolate (belonging to another species of CMG) as outgroup (Figure S4). The resulting ML trees were globally well supported (most bootstrap values >0.7) and appeared to be geographically structured. Interestingly, the historical ACMV genome sampled in 1928 in the Central African Republic clustered within a clade composed of modern isolates from the same country in both the ACMV DNA-A and DNA-B trees.

**Figure 4:**
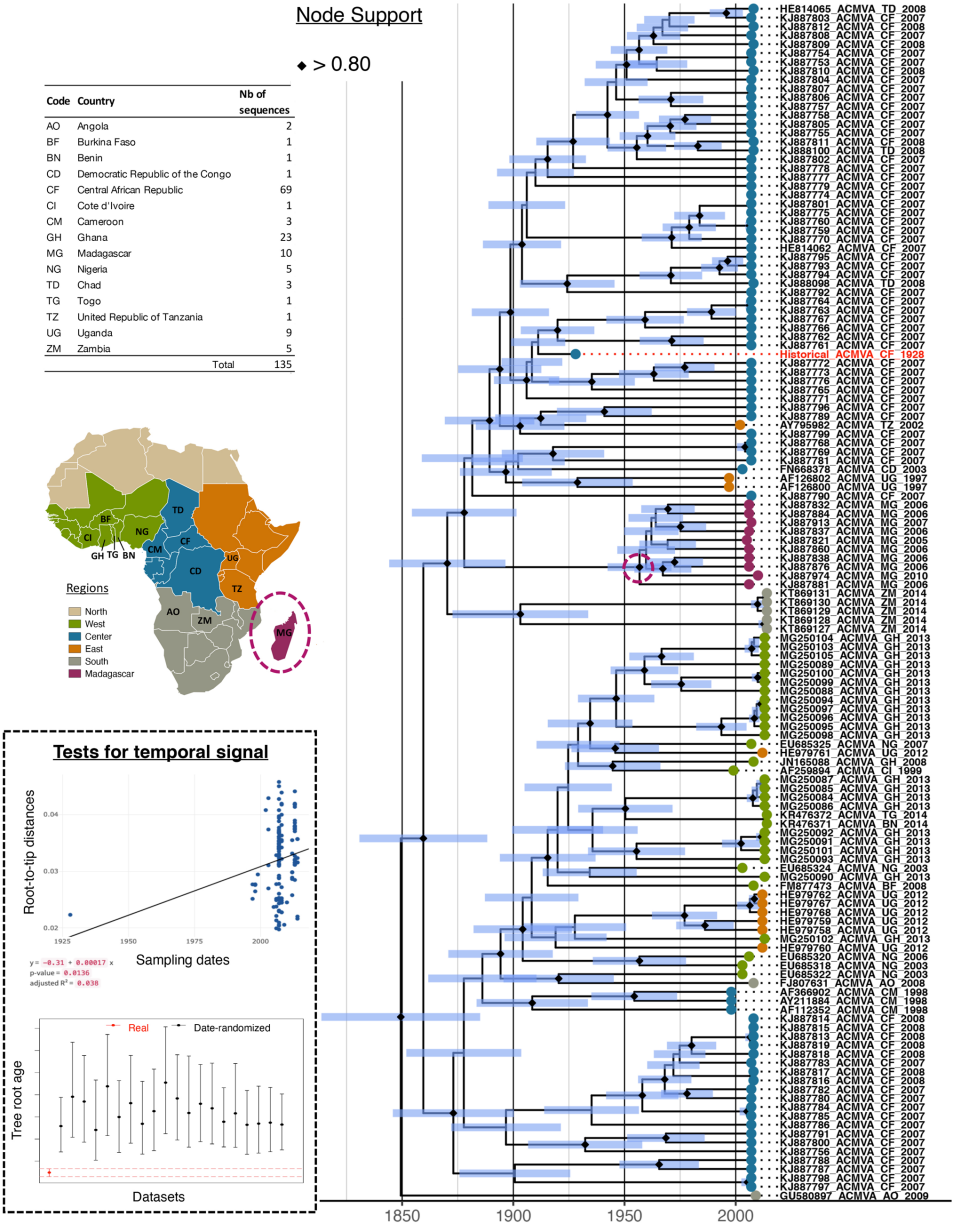
Bayesian dated tree of 134 sequences of ACMV DNA-A built from 1081 non-recombining SNPs. The historical DNA-A sequence is highlighted in red. Node support values with posterior probabilities above 0.8 are displayed by black diamonds. Node bars cover 95% Highest Probability Density of node height. Tips are colored according to the sample's geographic origin, according to the map on top left. The node corresponding to the common ancestor of all Malagasy isolates is circled in purple. Both tests of temporal signal (top: linear regression of root-to-tip distance on year of sampling date and bottom: date-randomization test) are presented in the dotted box.

In order to date the evolutionary history of ACMV, we used the ACMV DNA-A dataset, as the historical DNA-A sequence displayed a much higher depth and coverage than the ACMV DNA-B. As a prerequisite to perform tip-based calibration, we tested the presence of temporal signal in our tree with both a linear regression between sample ages and root-to-tip distance, and a date-randomisation test. Both statistical tests revealed the presence of a temporal signal (i.e. progressive accumulation of substitutions over time) within the ACMV DNA-A tree. The linear regression test displayed a significant positive slope (slope value= 0.00017, adjusted R^2^ = 0.0136 with a *p*-value = 0.038) and the date-randomisation test of the inferred root age of the real versus date-randomised dataset showed no overlap (Fig. 4). Additionally, our results showed no evidence of confounding between temporal and genetic structures (Mantel test: r = 0.001, p-value = 0.481), suggesting that the temporal signal detected is reliable and robust^51^. We therefore built a time-calibrated tree with BEAST^52^, which was globally congruent with the ML tree (similar topology and node supports; Figure 4). As in the ML-tree, the historical ACMV DNA-A sequence clustered within a clade composed of modern isolates sampled in the Central African Republic. This observation emphasises the value of historical samples in improving our understanding of the epidemiology of crop pathogens^53^. Indeed, our historical ACMV genome constitutes “fossil” evidence that CMD has occurred in the Central African Republic since at least 1928, consistently with the very first historical report of a disease resembling CMD that was made in this country in 1924^54^.

We inferred that the most recent common ancestor (MRCA) of all the analysed African ACMV DNA-A isolates most likely existed in 1849 [95% HPD: 1810–1880], a date that predates by more than 100 years the estimate of 1980 [95% HPD: 1990–1975] obtained by De Bruyn et al.^41^. The earlier estimate of the ACMV MRCA is more consistent with historical descriptions of the disease. Indeed, the earliest report of CMD-like symptoms in Africa was made in 1894 in what is now Tanzania^40^. Subsequent reports were made in the 1920s in relation to CMD epidemics in Sierra Leone, Ivory Coast, Ghana, Nigeria, Madagascar and Uganda^40^. By the end of the 1930s, CMD was reported from virtually all cassava-growing regions of the African mainland and surrounding islands.

We estimated a mean ACMV DNA-A substitution rate of 1.27x10^-4^ [95% HPD: 0.8 $\times$ 10^−4^–1.7 $\times$ 10^−4^] per site per year, with a standard deviation for the uncorrelated log-normal relaxed clock of 0.26 [95% HPD: 0.18–0.33], suggesting low substitution-rate heterogeneity amongst branches. This rate estimate is ~20 $\times$ and ~12.5 $\times$ lower than that the ones previously obtained using modern isolates only of ACMV^41^ and EACMV^55^, respectively.

**Figure 5:**
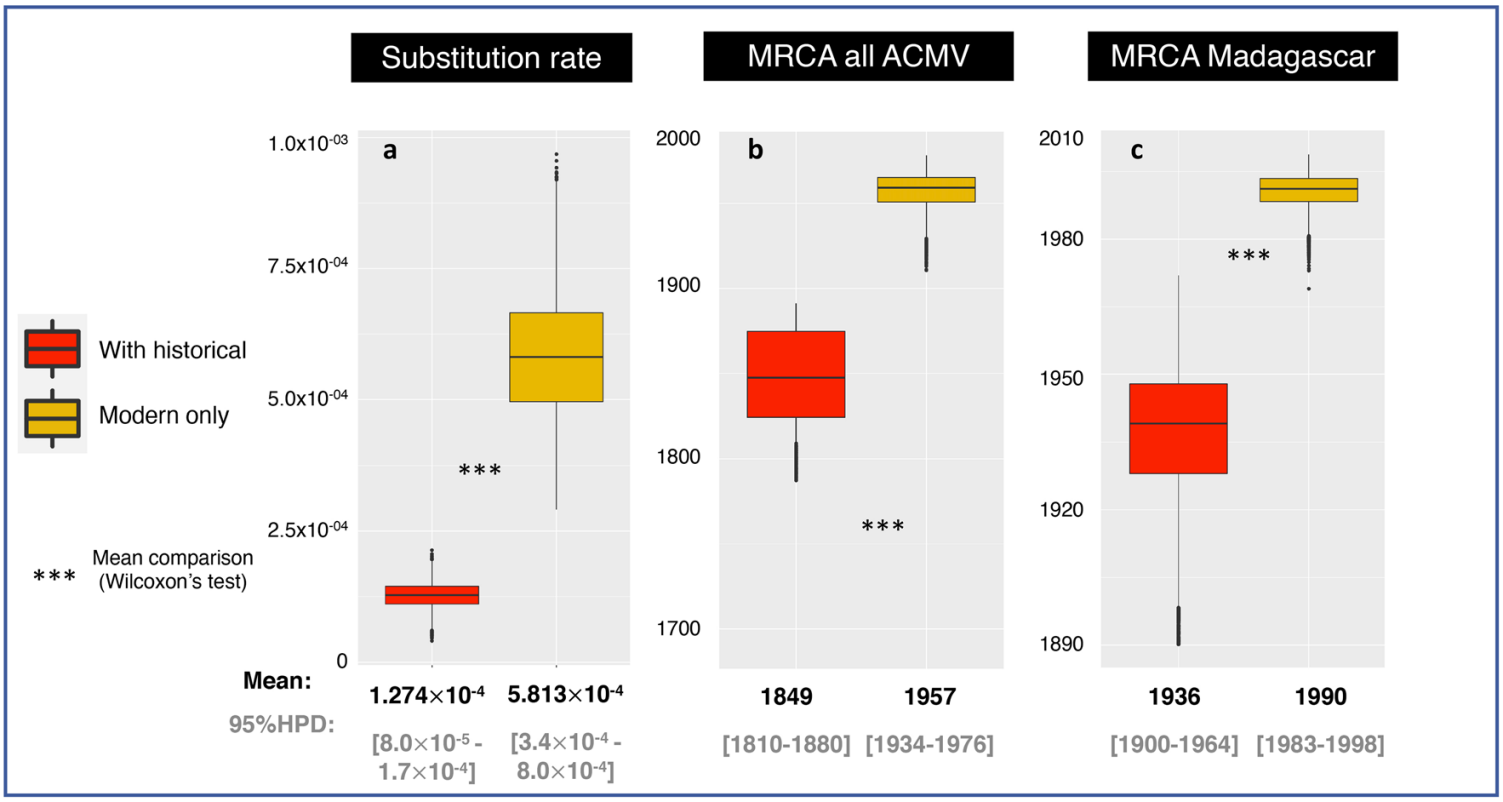
Bayesian estimations performed with or without including the historical genome. Substitution rate (**a**), MRCA of all (**b**) and from Madagascar (**c**) isolates, inferred with (red boxplot) and without (orange boxplot) the historical ACMV DNA-A component. ***p<0.001.

Although our reconstructed evolutionary history of ACMV appears broadly inconsistent with the latter study using only modern isolates, the two analyses are not directly comparable because of differences in dataset composition and other methodological choices. To specifically evaluate the contribution of the historical ACMV DNA-A sequence to ACMV DNA-A MRCA date and substitution rate estimates, we reanalysed our dataset after removing the historical sequence. As anticipated, this reanalysis under the exact same parameters still yielded significantly different results, while belonging to the same order of magnitude. Excluding the historical sequence yielded a five times higher substitution rate estimate (Fig. 5A). The standard deviation of substitution rates amongst branches for the uncorrelated log-normal relaxed clock did not change significantly from the analysis including the historical sequence (not shown). Excluding the historical sequence also yielded a significantly later estimate date for the MRCA of the analysed ACMV DNA-A sequences (1957 [95% HPD: 1934–1976], Fig. 5B). Similarly, the MRCA age for Malagasy island isolates (believed to have arisen from a single introduction) was estimated to 1936 [95% HPD: 1900 – 1964] and 1990 [95% HPD: 1983–1998] when including or excluding it, respectively (Fig. 5C).

The timeline of ACMV DNA-A evolution that we have inferred when including the historical sequence is likely to be more accurate than that determined without this sequence for two main reasons. First, this estimated timeline fits better with historical reports of CMD disease, dating back to 1894 in Africa and to the 1930s in Madagascar^40^. Second, the 95% credibility intervals of the estimated date of the ACMV DNA-A MRCA that was inferred without the historical sequence excludes 1928 and it therefore cannot be reconciled with the fact that a sequence sampled in 1928 clusters within the ACMV tree (i.e. it is not an outgroup) (Figure S5). Such striking lower substitution rate and hence higher divergence time estimates, when including ancient viral genome sequences, have been previously described in molecular dating studies focusing on different virus group representatives: barley stripe mosaic virus (BSMV)^17^, Human immunodeficiency virus^56^, hepatitis B virus^57^, as well as parvovirus B19^58^ (a ssDNA Baltimore group II virus to whom ACMV belongs), as recently reviewed in^15^.

In summary, our results illustrate that high-quality historical genomes of DNA viruses can be both reconstructed by sequencing the small RNA fraction of a plant herbarium specimen, harbouring siRNA characteristics and authenticated by analysing post-mortem RNA damage patterns. Such historical genomes represent “fossil” records of past viral diversity that have the potential to shed light on the spatiotemporal history of plant diseases. Indeed, our results demonstrate that CMD-causing ACMV variants were already present in the Central African Republic in 1928, supporting the accuracy of the description of a historical record of CMD made in 1924 from visual inspection of cassava leaves. Second, phylogenetic inferences performed with the inclusion of our historical ACMV DNA-A sequence significantly altered the inferred date at which the MRCA of all currently sampled ACMV variants likely existed, providing a better fit with historical reports than previous estimates and yielding a lower rate of ACMV DNA-A molecular evolution. Future studies including additional historical ACMV genome sequences that are more geographically/temporally dispersed will help us to refine the evolutionary parameters inferred herein. The presence of ACMV should also be tested in other herbarium plant species/families if one aims to investigate possible host-switching events that may have led to the emergence of CMD in cassava. More generally, similar investigations on other important viral crop pathogens will improve disease monitoring and sustainable control, while highlighting the importance of natural history collections.

## Material and methods

### Herbarium sampling

In 2014, the historical collection of cassava specimens of the National Herbarium of the Muséum National d'Histoire Naturelle, Paris (https://www.mnhn.fr/en) was searched for in 2014 for leaves displaying symptoms of CMD. Sample P04808771 (Fig. 1), a *Manihot glaziovii* specimen collected by C. Tisserant at Bambari, Central African Republic in 1928, displayed chlorotic mosaic and leaf distortion, two typical symptoms of CMD. A small leaf fragment (≈1cm^2^ / 12mg of dry material) was excised from this specimen using a disinfected blade and gloves, sealed in a clean envelope, transported to Reunion Island and stored in a vacuum-sealed box at 14°C until use. Permission to sample and perform destructive analysis on historical specimen P04808771 was obtained from the Muséum national d'Histoire naturelle (Paris, France). Collection of any plant material used in this study complies with institutional, national, and international guidelines.

### DNA extraction, amplification and sequencing

DNA isolation was performed in a bleach-cleaned molecular biology laboratory at the Centre Hospitalier Universitaire Sud Réunion that met the authenticity criteria for the extraction of ancient biomolecules^59^: a laboratory in which no plant samples had been manipulated before. Total DNA was extracted from the herbarium sample following manufacturer’s instructions of the Qiagen DN easy plant kit. We attempted to detect both viral and ACMV specific DNA using the standard RCA-Cloning-Sanger sequencing protocol^60^ and amplification of overlapping ACMV-specific PCR amplicons (ranging from 54 to 381nt), using validated primers (Harimalala, personal communication) listed in Table S2, respectively.

### RNA extraction, library preparation, sequencing and quality control

RNA isolation was also performed at the Centre Hospitalier Universitaire Sud Réunion. Total RNA was extracted from the herbarium sample using a PureLink Plant RNA Reagent kit (Ambion) and quantified using an Agilent 2200 Tapestation system (Agilent, France). Purification of siRNA, library preparation and sequencing were carried out by Fasteris NGS service team in Geneva, Switzerland. Using polyacrylamide gel electrophoresis, fragments of 18-30nt long were selected and converted into sequencing library using the Illumina TruSeq Small RNA Library Preparation kit. Sequencing was performed in a 1×50 cycle mode on a HiSeq instrument. Adaptors were trimmed from raw reads using the Illuminaclip option in Trimmomatic 0.36^61^. Additional quality-trimming was performed using the same tool to remove low Illumina quality score-associated bases (SLIDINGWINDOW:5:20) and reads shorter than 15nt (MINLEN:15). Those of size 18-30nt were retained as clean reads.

### Virus detection and taxonomic classification

To identify viruses from our historical sample, we first used VirusDetect^62^, a bioinformatic pipeline built to efficiently analyse large-scale small RNA (sRNA) datasets. We fixed all parameters to their default values and used the Sept 2019 GenBank reference virus genome database. In a second step, we used the dedicated short read aligner BWA-aln^63^ (with the following optimised options fixed as in VirusDetect pipeline: -n 1 -o 1 -e 1 -i 0 -l 15 -k 1) to map quality-trimmed reads to both viral (i.e. the species detected by VirusDetect) and host plant (*Manihot glaziovii* specimen GIShi—SRA: SRS597345) reference genomes. Our reads-mapping strategy was further assessed for the three following aspects. First, we evaluated the performance of another short-read aligner, Bowtie^64^, allowing one mismatch. Second, we compared the effect of mapping reads either independently or simultaneously to both ACMV DNA-A and DNA-B segments, in order to evaluate the influence of shared genomic regions. Finally, we assessed the effect of reference choice on mapping statistics and variant calling/filtering. To this aim, reads were mapped to three supplementary reference sequences (selected for their close, intermediate and distant phylogenetic proximity with the historical genome).

### Historical viral genome authentication and reconstruction

We examined the sequences for cytosine deamination patterns—a typical proxy of postmortem RNA damage–to authenticate the historical nature of the siRNA ACMV sequences obtained. Distributions of C to U *vs* other transitions along the siRNA reads were assessed from raw untrimmed reads using the dedicated mapDamage2 tool^65^. Postmortem RNA damage was compared between the historical specimen and RNA isolated from an ACMV infected *Manihot esculenta* leaf sample collected in Madagascar in 2017. The modern RNA sample was obtained using the exact same wet-lab protocol used to obtain RNA from the 1928 sample. Quality scores of post-mortem damaged bases were downscaled using the rescale parameter to correct for the effect of deamination and avoid generating artifactual SNPs in subsequent analyses. Historical ACMV DNA-A and DNA-B sequences were reconstructed from rescaled-BWA-aln generated BAM files for both DNA-A (JX658682) and DNA-B (KJ887590) GeneBank segment references. In brief, PCR duplicates were removed using picardtools 2.7.0 MarkDuplicates^66^ and depth statistics were computed with the genomecov option of BEDTools 2.24.0^67^, which were then graphically represented with CIRCOS 0.69.9^68^. SNPs were called with GATK UnifiedGenotyper^69^ and filtered out when their sequenced depth was <10 or their allelic frequency was < 0.6. Consensus historical sequences were then reconstructed by editing the reference DNA-A and DNA-B sequences with the remaining high-quality SNPs while replacing both filtered-out variants and unsequenced nucleotide sites (i.e. sites with a sequencing depth= 0) with “Ns”. Genes coding for AC3 and AC4 were deduced from other known ACMV sequences; all sequences were checked for open reading frame features.

In order to investigate the persistence of endogenous geminiviral sequences (EGSs) within *Manihot glaziovii* genomes, we downloaded raw reads of the two only available African *M. glaziovii* samples^47^ at the date of search (01/08/2021) within the SRA database (SRR2847420 & SRR2847424). After *de novo* assembly of the reads into contigs with SPAdes V3.15.2^70^ using default parameters, all reconstructed contigs were blasted (using BLASTN) on a custom-built database containing all described species of cassava mosaic geminiviruses. We predicted that the identification of chimeric contigs (composed of both cassava and virus sequences) would indicate the presence of EGSs. Instead, the detection of contigs displaying hits with virus sequences on their whole length would suggest plant infection by episomal viruses. Finally, the absence of any hits would reveal the absence of viral DNA, both from episomal and integrated forms, within *M. glaziovii* genomes.

### Phylogenetic inferences using both historical and modern sequences

Alignments of our historical ACMV DNA-A and DNA-B components with 134 (for DNA-A) and 99 (for DNA-B) publicly available ACMV genome component sequences sampled between 1978 and 2014 (Table S4) were constructed with MAFFT^71^ for phylogenetic analyses. Each of these alignments also included a CMMGV sequence as an outgroup (accession number HE617299 and HE617300 for DNA-A and DNA-B, respectively). Regions acquired via recombination were identified with RDP4^50^ with default settings. Events that were detected by three or more methods with P-values <0.05 were accepted as credible and removed to avoid the potentially biasing impacts of recombination on phylogenetic reconstruction. Note that the historical sequence was analysed with particular scrutiny and recombination events detected with a single method were taken into account. Maximum likelihood trees for each of these alignments were constructed using RAxML 8.2.4^72^ using a rapid bootstrap test and the GTR+G+I model of nucleotide substitution was chosen as best-fitted model based on the Bayesian Information Criterion (BIC) computed with JModelTest2.0^73^.

The existence of a temporal signal in this dataset was investigated using two different tests. First, a linear regression was fitted between sample age and root-to-tip distance using the distRoot function of the adephylo R package^74^. Temporal signal was considered present if a significant positive correlation was observed. Secondly, we performed a date-randomisation test (DRT)^75^ with 20 independent date-randomised datasets using the R package, TipDatingBeast^76^. Temporal signal was considered present when there was no overlap between the inferred root height 95% highest posterior density (95% HPD) of the initial dataset and that of 20 date-randomised datasets. Finally, we also investigated whether our dataset showed confounding effects between temporal and genetic structures using a Mantel confounding test which investigate whether closely related sequences were more likely to have been sampled at similar times. This additional test is important because both the root-to-tip regression and the DRT can be confounded in such a situation^51^.

Tip-dating was performed with BEAST 1.8.4^52^ considering a GTR substitution model with a Γ distribution and invariant sites (GTR+G+I) along with an uncorrelated log-normal relaxed (UCLNR) clock to account for minor variations between lineages. Bayes factors calculated from the marginal likelihoods using both path and stepping-stone sampling methods shown “very strong” support (BF>10^77^) for UCLNR over strict (S) and random local (RL) clocks. To minimise prior assumptions about demographic history, an extended Bayesian skyline plot (EBSP) approach was adopted to integrate data over different coalescent histories^78^. Three independent chains were run for 25 million steps and sampled every 2500 steps with a burn-in of the first 2500 steps. Convergence to the stationary distribution and sufficient sampling and mixing were checked by inspection of posterior samples (effective sample size >200) in Tracer 1.7.1^79^. Parameter estimation was based on the samples combined from the different chains. The best-supported tree was estimated from the combined samples using the maximum clade credibility method implemented in TreeAnnotator. In order to specifically assess the effect of including our historical genome in the dating calibration, we computed the same inferences on a dataset where the 1928 DNA-A sequence was excluded (i.e. using only sequences sampled after 1977). Wilcoxon rank sum tests with continuity correction were performed to compare the means of the posterior estimates obtained from both datasets.

## Supplementary Information

Supplementary Information.

## Data Availability

Raw reads were deposited to the Sequence Read Archive (SRR13608699). Consensus historical genome reconstructed for ACMV DNA-A and DNA-B molecules have also been deposited on GenBank database (MW788219 & MW788220). The modern genomes used in this study have previously been published in the NCBI GenBank repository under accession numbers listed in Table S4.
