# Supplementary material for: Contribution of historical herbarium small RNAs to the reconstruction of a cassava mosaic geminivirus evolutionary history
Source: Sci Rep. 2021 Oct 28;11:21280. doi: 10.1038/s41598-021-00518-w (PMC8553777; doi:10.1038/s41598-021-00518-w)
Supplement: Supplementary file 1 — Supplementary Information. [file 41598_2021_518_MOESM1_ESM.docx]

**Supplementary data**

**Contribution of historical herbarium small RNAs to the reconstruction of a cassava mosaic geminivirus evolutionary history**

Adrien Rieux^1*^, Paola Campos^1,2^, Arnaud Duvermy^1^, Sarah Scussel^1^, Darren Martin^3^, Myriam Gaudeul^2^, Pierre Lefeuvre^1^, Nathalie Becker^2^, Jean-Michel Lett^1*^

**Figure S1**. Virus Detect results showing coverage statistics of both reconstructed ACMV DNA-A and DNA-B reference sequences along with a reference sequence schematic representation (blue line) and the mapping of contigs reconstructed from small RNA reads (red line).

1) Comparison of mapping statistics obtained with both BWA-aln and Bowtie short-read aligners. Left: % of endogenous DNA, depth and coverage. Right: Venn diagram showing the number of shared and specific mapped reads. GeneBank segment references: DNA-A (JX658682) and DNA-B (KJ887590).

2) Comparison of mapping statistics obtained by mapping reads either simultaneously or independently to both DNA-A and DNA-B segment references. % of endogenous DNA, depth and coverage. GeneBank segment references: DNA-A (JX658682) and DNA-B (KJ887590). Mapper: BWA-aln.

3) Comparison of mapping statistics and pairwise nucleotide identity between historical consensus sequences obtained by mapping (using BWA-aln) reads to different reference genomes (left). Zoom within ACMV DNA-A (green) & DNA-B (red) ML trees built with all modern sequences and the consensus historical genomes reconstructed using different reference sequences (right).

**Figure S2.** Assessment of our small reads mapping strategy

**Figure S3.** ACMV DNA-A and DNA-B coverage by small RNA sequence reads. Genomic organization of open reading frames (protein and gene names) are indicated on corresponding arrows, pointing leftwards when on complementary strand. IR: conserved intergenic region (dotted line). ACMV-A: CP, coat protein; MP movement protein; Rep, replication-associated protein; TrAP, transcriptional-activator protein; Ren, replication enhancer protein. ACMV-B: NSP, nuclear-shuttle protein; MP, movement protein.

**Figure S4**. ML tree reconstructed from both the ACMV DNA-A (left) and ACMV DNA-B (right) sequences alignments, using a CMMGV isolate as outgroup (which has been removed from the above plot). Historical ACMV components are highlighted in red and tip colours are associated with regions of sampling.

**Figure S5**. Bayesian dated tree of 133 modern sequences of ACMV DNA-A (excluding the historical genome, which expected branching is indicated with the red star symbol). The vertical red dotted line indicates the year of isolation of the historical sample.

**Table S1:** Summary of the recombination events detected by RDP4. The methods used to detect recombination are RDP (R) GENCONV (G), BOOTSCAN (B), MAXCHI (M), CHIMERA (C), SISCAN (S) and 3SEQ (T). The method with the highest P-value for each recombination event is bolded.

**Table S2: PCR primers used to attempt amplifying historical viral DNA.**

**Table S3:** Blasting *M. glaziovii* contigs to a database containing all described species of cassava mosaic geminiviruses

To produce the above table, we downloaded raw reads of the two only available African *M. glaziovii* samples at the date of search (01/08/2021) within the SRA database (SRR2847420 & SRR2847424). After de novo assembly of the reads into contigs, all reconstructed contigs were blasted on a custom-built database containing all described species of cassava mosaic geminiviruses. Of the contigs that displayed similarities with geminiviruses (listed in the query column) all best hits were obtained with EACMV and covered 99 to 100% of the contigs. No chimeric contigs (containing both viruses and cassava sequences, that would indicate the presence of EGS), were detected, suggesting that the analysed *M. glaziovii* genomes were generated from plants contaminated with episomal geminiviruses and do not show any evidence of EGSs.

**Table S4:** List of public ACMV sequences (DNA-A and -B) used during the course of this study

| **Accession nb** | **Sequence** | **Sampling year** | **Geographic origin** |
| --- | --- | --- | --- |
| HE617299 | CMMGVA (outgroup) | 2012 | Madagascar |
| FJ807631 | ACMVA | 2008 | Angola |
| GU580897 | ACMVA | 2009 | Angola |
| GU580897 | ACMVA | 2009 | Angola |
| FM877473 | ACMVA | 2008 | Burkina Faso |
| KR476371 | ACMVA | 2014 | Benin |
| FN668378 | ACMVA | 2003 | Democratic Republic of the Congo |
| KJ887768 | ACMVA | 2007 | Central African Republic |
| KJ887769 | ACMVA | 2007 | Central African Republic |
| KJ887789 | ACMVA | 2007 | Central African Republic |
| KJ887796 | ACMVA | 2007 | Central African Republic |
| KJ887779 | ACMVA | 2007 | Central African Republic |
| KJ887792 | ACMVA | 2007 | Central African Republic |
| KJ887794 | ACMVA | 2007 | Central African Republic |
| KJ887793 | ACMVA | 2007 | Central African Republic |
| KJ887795 | ACMVA | 2007 | Central African Republic |
| HE814062 | ACMVA | 2007 | Central African Republic |
| KJ887770 | ACMVA | 2007 | Central African Republic |
| KJ887774 | ACMVA | 2007 | Central African Republic |
| KJ887775 | ACMVA | 2007 | Central African Republic |
| KJ887801 | ACMVA | 2007 | Central African Republic |
| KJ887759 | ACMVA | 2007 | Central African Republic |
| KJ887760 | ACMVA | 2007 | Central African Republic |
| KJ887765 | ACMVA | 2007 | Central African Republic |
| KJ887776 | ACMVA | 2007 | Central African Republic |
| KJ887772 | ACMVA | 2007 | Central African Republic |
| KJ887773 | ACMVA | 2007 | Central African Republic |
| KJ887771 | ACMVA | 2007 | Central African Republic |
| KJ887766 | ACMVA | 2007 | Central African Republic |
| KJ887767 | ACMVA | 2007 | Central African Republic |
| KJ887763 | ACMVA | 2007 | Central African Republic |
| KJ887764 | ACMVA | 2007 | Central African Republic |
| KJ887761 | ACMVA | 2007 | Central African Republic |
| KJ887762 | ACMVA | 2007 | Central African Republic |
| KJ887778 | ACMVA | 2007 | Central African Republic |
| KJ887802 | ACMVA | 2007 | Central African Republic |
| KJ887811 | ACMVA | 2008 | Central African Republic |
| KJ887755 | ACMVA | 2007 | Central African Republic |
| KJ887805 | ACMVA | 2007 | Central African Republic |
| KJ887758 | ACMVA | 2007 | Central African Republic |
| KJ887757 | ACMVA | 2007 | Central African Republic |
| KJ887806 | ACMVA | 2007 | Central African Republic |
| KJ887807 | ACMVA | 2007 | Central African Republic |
| KJ887804 | ACMVA | 2007 | Central African Republic |
| KJ887810 | ACMVA | 2008 | Central African Republic |
| KJ887753 | ACMVA | 2007 | Central African Republic |
| KJ887754 | ACMVA | 2007 | Central African Republic |
| KJ887808 | ACMVA | 2007 | Central African Republic |
| KJ887812 | ACMVA | 2008 | Central African Republic |
| KJ887803 | ACMVA | 2007 | Central African Republic |
| KJ887809 | ACMVA | 2008 | Central African Republic |
| KJ887777 | ACMVA | 2007 | Central African Republic |
| KJ887781 | ACMVA | 2007 | Central African Republic |
| KJ887799 | ACMVA | 2007 | Central African Republic |
| KJ887790 | ACMVA | 2007 | Central African Republic |
| KJ887786 | ACMVA | 2007 | Central African Republic |
| KJ887784 | ACMVA | 2007 | Central African Republic |
| KJ887785 | ACMVA | 2007 | Central African Republic |
| KJ887816 | ACMVA | 2008 | Central African Republic |
| KJ887817 | ACMVA | 2008 | Central African Republic |
| KJ887780 | ACMVA | 2007 | Central African Republic |
| KJ887782 | ACMVA | 2007 | Central African Republic |
| KJ887818 | ACMVA | 2008 | Central African Republic |
| KJ887819 | ACMVA | 2008 | Central African Republic |
| KJ887813 | ACMVA | 2008 | Central African Republic |
| KJ887814 | ACMVA | 2008 | Central African Republic |
| KJ887815 | ACMVA | 2008 | Central African Republic |
| KJ887783 | ACMVA | 2007 | Central African Republic |
| KJ887756 | ACMVA | 2007 | Central African Republic |
| KJ887791 | ACMVA | 2007 | Central African Republic |
| KJ887800 | ACMVA | 2007 | Central African Republic |
| KJ887797 | ACMVA | 2007 | Central African Republic |
| KJ887798 | ACMVA | 2007 | Central African Republic |
| KJ887787 | ACMVA | 2007 | Central African Republic |
| KJ887788 | ACMVA | 2007 | Central African Republic |
| AF259894 | ACMVA | 1999 | Cote d'Ivoire |
| AF112352 | ACMVA | 1998 | Cameroon |
| AF366902 | ACMVA | 1998 | Cameroon |
| AY211884 | ACMVA | 1998 | Cameroon |
| JN165088 | ACMVA | 2008 | Ghana |
| MG250086 | ACMVA | 2013 | Ghana |
| MG250084 | ACMVA | 2013 | Ghana |
| MG250085 | ACMVA | 2013 | Ghana |
| MG250087 | ACMVA | 2013 | Ghana |
| MG250098 | ACMVA | 2013 | Ghana |
| MG250095 | ACMVA | 2013 | Ghana |
| MG250094 | ACMVA | 2013 | Ghana |
| MG250097 | ACMVA | 2013 | Ghana |
| MG250096 | ACMVA | 2013 | Ghana |
| MG250088 | ACMVA | 2013 | Ghana |
| MG250099 | ACMVA | 2013 | Ghana |
| MG250100 | ACMVA | 2013 | Ghana |
| MG250089 | ACMVA | 2013 | Ghana |
| MG250104 | ACMVA | 2013 | Ghana |
| MG250105 | ACMVA | 2013 | Ghana |
| MG250103 | ACMVA | 2013 | Ghana |
| MG250090 | ACMVA | 2013 | Ghana |
| MG250101 | ACMVA | 2013 | Ghana |
| MG250091 | ACMVA | 2013 | Ghana |
| MG250092 | ACMVA | 2013 | Ghana |
| MG250102 | ACMVA | 2013 | Ghana |
| MG250093 | ACMVA | 2013 | Ghana |
| KJ887860 | ACMVA | 2006 | Madagascar |
| KJ887913 | ACMVA | 2007 | Madagascar |
| KJ887837 | ACMVA | 2006 | Madagascar |
| KJ887832 | ACMVA | 2006 | Madagascar |
| KJ887884 | ACMVA | 2006 | Madagascar |
| KJ887974 | ACMVA | 2010 | Madagascar |
| KJ887838 | ACMVA | 2006 | Madagascar |
| KJ887876 | ACMVA | 2006 | Madagascar |
| KJ887881 | ACMVA | 2006 | Madagascar |
| KJ887821 | ACMVA | 2005 | Madagascar |
| EU685324 | ACMVA | 2003 | Nigeria |
| EU685325 | ACMVA | 2007 | Nigeria |
| EU685318 | ACMVA | 2003 | Nigeria |
| EU685320 | ACMVA | 2006 | Nigeria |
| EU685322 | ACMVA | 2003 | Nigeria |
| KJ888098 | ACMVA | 2008 | Chad |
| KJ888100 | ACMVA | 2008 | Chad |
| HE814065 | ACMVA | 2008 | Chad |
| KR476372 | ACMVA | 2014 | Togo |
| AY795982 | ACMVA | 2002 | United Republic of Tanzania |
| AF126800 | ACMVA | 1997 | Uganda |
| AF126802 | ACMVA | 1997 | Uganda |
| HE979760 | ACMVA | 2012 | Uganda |
| HE979761 | ACMVA | 2012 | Uganda |
| HE979768 | ACMVA | 2012 | Uganda |
| HE979762 | ACMVA | 2012 | Uganda |
| HE979767 | ACMVA | 2012 | Uganda |
| HE979758 | ACMVA | 2012 | Uganda |
| HE979759 | ACMVA | 2012 | Uganda |
| KT869129 | ACMVA | 2014 | Zambia |
| KT869130 | ACMVA | 2014 | Zambia |
| KT869131 | ACMVA | 2014 | Zambia |
| KT869127 | ACMVA | 2014 | Zambia |
| KT869128 | ACMVA | 2014 | Zambia |
| HE617300 | CMMGVB (outgroup) | 2012 | Madagascar |
| KR476373 | ACMVB | 2014 | Benin |
| FN668379 | ACMVB | 2003 | Democratic Republic of the Congo |
| KJ887632 | ACMVB | 2007 | Central African Republic |
| KJ887631 | ACMVB | 2007 | Central African Republic |
| KJ887630 | ACMVB | 2008 | Central African Republic |
| KJ887629 | ACMVB | 2008 | Central African Republic |
| KJ887628 | ACMVB | 2008 | Central African Republic |
| KJ887627 | ACMVB | 2008 | Central African Republic |
| KJ887626 | ACMVB | 2008 | Central African Republic |
| KJ887625 | ACMVB | 2008 | Central African Republic |
| KJ887624 | ACMVB | 2007 | Central African Republic |
| KJ887623 | ACMVB | 2007 | Central African Republic |
| KJ887622 | ACMVB | 2007 | Central African Republic |
| KJ887621 | ACMVB | 2007 | Central African Republic |
| KJ887620 | ACMVB | 2007 | Central African Republic |
| KJ887619 | ACMVB | 2007 | Central African Republic |
| KJ887618 | ACMVB | 2007 | Central African Republic |
| KJ887617 | ACMVB | 2007 | Central African Republic |
| KJ887616 | ACMVB | 2007 | Central African Republic |
| KJ887615 | ACMVB | 2007 | Central African Republic |
| KJ887614 | ACMVB | 2007 | Central African Republic |
| KJ887613 | ACMVB | 2007 | Central African Republic |
| KJ887612 | ACMVB | 2007 | Central African Republic |
| KJ887611 | ACMVB | 2007 | Central African Republic |
| KJ887610 | ACMVB | 2007 | Central African Republic |
| KJ887609 | ACMVB | 2007 | Central African Republic |
| KJ887608 | ACMVB | 2007 | Central African Republic |
| KJ887607 | ACMVB | 2007 | Central African Republic |
| KJ887606 | ACMVB | 2007 | Central African Republic |
| KJ887605 | ACMVB | 2007 | Central African Republic |
| KJ887604 | ACMVB | 2007 | Central African Republic |
| KJ887603 | ACMVB | 2007 | Central African Republic |
| KJ887602 | ACMVB | 2007 | Central African Republic |
| KJ887601 | ACMVB | 2007 | Central African Republic |
| KJ887600 | ACMVB | 2007 | Central African Republic |
| KJ887599 | ACMVB | 2007 | Central African Republic |
| KJ887598 | ACMVB | 2007 | Central African Republic |
| KJ887597 | ACMVB | 2007 | Central African Republic |
| KJ887596 | ACMVB | 2007 | Central African Republic |
| KJ887595 | ACMVB | 2007 | Central African Republic |
| KJ887594 | ACMVB | 2007 | Central African Republic |
| KJ887593 | ACMVB | 2007 | Central African Republic |
| KJ887592 | ACMVB | 2007 | Central African Republic |
| KJ887591 | ACMVB | 2007 | Central African Republic |
| KJ887590 | ACMVB | 2007 | Central African Republic |
| KJ887589 | ACMVB | 2007 | Central African Republic |
| KJ887588 | ACMVB | 2007 | Central African Republic |
| KJ887587 | ACMVB | 2007 | Central African Republic |
| KJ887586 | ACMVB | 2007 | Central African Republic |
| KJ887585 | ACMVB | 2007 | Central African Republic |
| KJ887584 | ACMVB | 2007 | Central African Republic |
| KJ887583 | ACMVB | 2007 | Central African Republic |
| KJ887582 | ACMVB | 2007 | Central African Republic |
| AF259895 | ACMVB | 1999 | Cote d'Ivoire |
| AF112353 | ACMVB | 1998 | Cameroon |
| JN165086 | ACMVB | 2008 | Ghana |
| HG530122 | ACMVB | 2012 | Kenya |
| HG530121 | ACMVB | 2012 | Kenya |
| HG530120 | ACMVB | 2012 | Kenya |
| HG530119 | ACMVB | 2012 | Kenya |
| HG530118 | ACMVB | 2012 | Kenya |
| HG530117 | ACMVB | 2012 | Kenya |
| KJ887744 | ACMVB | 2011 | Madagascar |
| KJ887743 | ACMVB | 2011 | Madagascar |
| KJ887741 | ACMVB | 2011 | Madagascar |
| KJ887739 | ACMVB | 2011 | Madagascar |
| KJ887710 | ACMVB | 2011 | Madagascar |
| KJ887707 | ACMVB | 2011 | Madagascar |
| KJ887706 | ACMVB | 2011 | Madagascar |
| KJ887705 | ACMVB | 2011 | Madagascar |
| KJ887692 | ACMVB | 2010 | Madagascar |
| KJ887681 | ACMVB | 2008 | Madagascar |
| KJ887651 | ACMVB | 2006 | Madagascar |
| KJ887638 | ACMVB | 2006 | Madagascar |
| KJ887637 | ACMVB | 2006 | Madagascar |
| KJ887636 | ACMVB | 2006 | Madagascar |
| KJ887634 | ACMVB | 2005 | Madagascar |
| KJ887752 | ACMVB | 2008 | Chad |
| KJ887751 | ACMVB | 2008 | Chad |
| KJ887750 | ACMVB | 2008 | Chad |
| KR476374 | ACMVB | 2014 | Togo |
| HE979796 | ACMVB | 2012 | Uganda |
| HE979795 | ACMVB | 2012 | Uganda |
| HE979794 | ACMVB | 2012 | Uganda |
| HE979793 | ACMVB | 2012 | Uganda |
| HE979792 | ACMVB | 2012 | Uganda |
| HE979791 | ACMVB | 2012 | Uganda |
| HE979790 | ACMVB | 2012 | Uganda |
| HE979789 | ACMVB | 2012 | Uganda |
| HE979788 | ACMVB | 2012 | Uganda |
| HE979787 | ACMVB | 2012 | Uganda |
| HE979786 | ACMVB | 2012 | Uganda |
| HE979785 | ACMVB | 2012 | Uganda |
| HE979784 | ACMVB | 2012 | Uganda |
| HE979783 | ACMVB | 2012 | Uganda |
| HE979782 | ACMVB | 2012 | Uganda |
| HE979781 | ACMVB | 2012 | Uganda |
| AF126803 | ACMVB | 1997 | Uganda |
| AF126801 | ACMVB | 1997 | Uganda |
